# Epidemiological and Clinical Features of Primary Giant Cell Tumors of the Distal Radium: A Multicenter Retrospective Study in China

**DOI:** 10.1038/s41598-017-09486-6

**Published:** 2017-08-22

**Authors:** Hongbin Cao, Fengsong Lin, Yongcheng Hu, Liming Zhao, Xiuchun Yu, Zhen Wang, Zhaoming Ye, Sujia Wu, Shibing Guo, Guochuan Zhang, Jinghua Wang

**Affiliations:** 1 Department of Foot and Ankle Surgery, Tianjin Hospital, 406 Jiefang South Road, Tianjin, 300210 P. R. China; 2 Department of Traumatology, Tianjin Hospital, 406 Jiefang South Road, Tianjin, 300210 P. R. China; 3 Department of Bone Tumor, Tianjin Hospital, 406 Jiefang South Road, Hexi District, Tianjin, 300210 P. R. China; 4 The Graduate School, Tianjin Medical University, 22 Qixiangtai Road, Heping District, Tianjin, 300071 P. R. China; 5 Department of Orthopedics, The General Hospital of Jinan Military Commanding Region, 25 Shifan Road, Jinan, Shandong 250031 P. R. China; 6 Department of Orthopedics, Xijing Hospital, Forth Military Medical University, No. 15 West Changle Road, Xincheng District, Xi’an, Shaanxi 710032 P. R. China; 7 Centre for Orthopaedic Research, Department of Orthopaedics, The Second Affiliated Hospital of Zhejiang University, School of Medicine, 88 Jiefang Road, Hangzhou, 310008 P. R. China; 8 Department of Orthopredics, Jin Ling Hospital, 305 Zhong Shan East Road, Nanjing, 210002 Jiangsu Province P. R. China; 9 Orthopedics Department, Second Affiliated Hospital of Inner Mongolia Medical University, 1 Yingfang Road, Huimin District, Hohhot, 010050 P. R. China; 10 Department of Oncology of Bone, The Third Hospital of Hebei Medical University, 139 Ziqiang Rd, Shijiazhuang, 050051 P. R. China; 11 Center of Clinical Epidemiology, Tianjin Medical University General Hospital, 154 Anshan Road, Heping District, Tianjin, 300052 P. R. China

## Abstract

Giant cell tumors of the distal radius are challenging for surgeons because they are associated with high recurrence rates and poor functional outcomes. Between June 2005 and October 2015, patients with primary giant cell tumors of the distal radius were recruited from seven orthopedic centers in China. The patients’ clinical features and demographic characteristics were obtained from medical records and reviewed retrospectively. Overall, 48 cases of giant cell tumors of the distal radius were assessed in this study. These patients were more likely to be between 20 and 40 years of age, to have a Campanacci grade of III, and to undergo a surgical style of resection. The prevalence of pathological fractures was 12.5% overall (20.0% in men and 4.3% in women). The prevalence of local recurrence was 30.0% overall (38.1% in men and 21.1% in women) during the average follow-up period of 62.5 months, with a pulmonary metastasis rate of 5.0%. Giant cell tumors of the distal radius were predominant in men and were more likely to recur locally than around the knee. These findings suggest that it is crucial to evaluate the optimal surgical approach for balancing local recurrence control and functional outcomes to reduce the disease burden.

## Introduction

Giant cell tumors (GCTs) are primary intramedullary bone tumors composed of mononuclear and giant mononuclear cells, resembling osteoclasts^1^. Mononuclear cells are the primary pathological feature of GCT, and they determine the biological behavior of GCTs. GCTs account for only 3–8% of primary bone tumors in Western populations, compared with 20% in Asian countries^2–8^.

GCTs most commonly occur among 20–40-year-olds and are likely to be located at many sites of body. However, they usually involve the end of a long bone, and more than half of them occur around the knee^3, 4, 9–12^. About 10% of GCTs undergo malignant transformation, and pulmonary metastases occur in 1% to 4% of cases^13^. It has been reported that the postoperative recurrence rate is 10–65%^14–16^. Previous studies have indicated that GCTs predominantly occur among women^17, 18^. However, some studies have shown a male predominance^19–22^.

The distal radius is the third most common site for GCTs to occur^7^, accounting for an average of 10% (8–13%) of GCT occurrences^23–28^. GCTs in this location are more likely to have a higher rate of local recurrence, to metastasize^29, 30^, and to have poor functional outcomes; thus, they are one of the most controversial bone tumors. The surgical management of GCTs of the distal radius remains controversial due to the epiphyseal nature of distal radius. However, all of the previous studies were performed in single institutions; reports on the epidemiological features of GCTs of the distal radius from multicenter studies are rare.

Thus, in this study, we aimed to assess the epidemiological and clinical characteristics of GCTs of the distal radius in China using data from the Giant Cell Tumor of China (GTOC), a synergistic, multicenter study of GCTs.

## Results

### Overall demographic and clinical characteristics

Of the 48 patients with GCT of the distal radius, 25 (52.1%) were men and 23 (47.9%) were women, with a male to female ratio of 1.1:1. Of these, 68.8% of cases occurred in patients between 20 and 40 years of age, 79.2% were Campanacci grade III, and 66.7% were treated with resection. The prevalence of pathological fractures was 12.5% (Table 1).

**Table 1:** The distribution characteristics of primary GCT of distal radius in China.

Characteristics	Number	Frequency
Total:	48	—
Men	25	52.1
Women	23	47.9
Age group, years		
<20	5	10.4
20~40	33	68.8
>40	10	20.8
Campanacci grade		
I	1	2.1
II	9	18.8
III	38	79.2
Side:		
Left	24	50.0
Right	24	50.0
Fracture	6	12.5
Dislocation	3	6.3
Surgical style:		
Curage	16	33.3
Resection	32	66.7

### Clinical characteristics by sex

Table 2 shows that the me﻿an ages at first diagnosis of GCT of the distal radius were 30.6 years overall (28.7 years in men and 32.6 years in women). Female patients with GCT of the distal radius were more likely than men to have a GCT that was Campanacci grade III (91.3% vs. 68.0%), on the right (56.5% vs. 44.0%), and treated with resection (73.9% vs. 60.0%), although the differences between women and men did not reach statistical significance (all P > 0.05). Simultaneously, there was higher frequency in men than in women aged ≤40 years (84.0% vs. 73.9%, P = 0.487), with pathological fractures (20.0% vs. 4.3%, P = 0.191), and with dislocation (12.0% vs. 0%, P = 0.235).

**Table 2:** Clinical characteristics of primary GCT of the distal radius by gender.

Characteristics	Total	Man	Women	P
﻿﻿Age, means (SD), years	30.6	28.7	32.6	0.219
﻿﻿Age group, n (%)				0.616
<20 years	5 (10.4)	4 (16.0)	1 (4.3)	
20~40 years	33 (68.8)	17 (68.0)	16 (69.6)	
>40 years	10 (20.8)	4 (16.0)	6 (26.1)	
Campanacci grade, n (%)				0.127
I	1 (2.1)	1 (4.0)	0	
II	9 (18.8)	7 (28.0)	2 (8.7)	
III	38 (79.2)	17 (68.0)	21 (91.3)	
Side, n (%)				0.386
Left	24 (50)	14 (56.0)	10 (43.5)	
Right	24 (50)	11 (44.0)	13 (56.5)	
Fracture, n (%)	6 (12.5)	5 (20.0)	1 (4.3)	0.191
Dislocation, n (%)	3 (6.3)	3 (12.0)	0	0.235
Surgical style, n (%)				0.307
Curage	16 (33.3)	10 (40.0)	6 (26.1)	
Resection	32 (66.7)	15 (60.0)	17 (73.9)	

### Clinical characteristics by age group

There were no remarkable age differences in clinical features, but a slightly higher frequency of GCTs occurred in patients >40 years of age than in patients ≤40 years of age for Campanacci grade III (100% vs. 73%) and pathological fractures (18.2% vs. 10.8%). Conversely, patients ≤40 years of age were more likely than patients aged >40 to have a dislocation (18.1% vs. 0; Table 3).

**Table 3:** Clinical characteristics of primary GCT of the distal radius by age group.

Characteristics	Total	≤40 years	>40 years	P
Gender, n (%)				0.487
Man	25 (52.1)	21 (84.0)	4 (16.0)	
Women	23 (47.9)	17 (73.9)	6 (26.1)	
Campanacci grade, n (%)				0.153
I	1 (2.1)	1 (2.7)	0	
II	9 (18.8)	9 (24.3)	0	
III	38 (79.2)	27 (73.0)	11 (100.0)	
Side, n (%)				0.731
Left	24 (50)	18 (48.6)	6 (54.5)	
Right	24 (50)	19 (51.4)	5 (45.5)	
Fracture, n (%)	6 (12.5)	4 (10.8)	2 (18.2)	0.609
Dislocation, n (%)	3 (6.3)	3 (18.1)	0	1.000
Surgical style, n (%)				0.293
Curage	16 (33.3)	14 (37.8)	2 (18.2)	
Resection	32 (66.7)	23 (62.2)	9 (81.8)	

### Recurrence and metastasis

During a median follow-up period of 62.5 months, 12 patients out of 40 who were qualified for follow-up developed a local recurrence, for a rate of 30% overall. The frequency of local recurrence was 38.1% for men and 21.1% for women. Moreover, pulmonary metastasis was observed in two cases (1 man and 1 woman), for an overall rate of 5.0% (4.8% in men and 5.3% in women).

## Discussion

This is the first large-sample report of the epidemiological and clinical features of patients with GCT of the distal radius from a multicenter study in China.

GCTs of the distal radius have been established to be the most common type of GCT after that of the distal femur and of the proximal tibia^7, 31, 32^. A female predominance for GCTs of the distal radius has been reported in previous studies^33–35^; a male-to-female ratio of 0.4:1 was reported in Iran^33^, of 0.7:1 in Beijing, China^34^, and of 0.5:1 in Hong Kong, China^35^. However, an inverse male predominance for GCTs of the distal radius has been observed in other studies, with reported ratios of 1.3:1^36^ and 1.4:1^37^. Consistent with these studies, we found a male predominance in GCT of the distal radius, with a male-to-female ratio of 1.1:1 in this study.

GCTs usually occur in young adults aged 20–40 years, accounting for 70–80% of all GCT patients. The mean age at diagnosis of GCT has been presented as being between 29 years and 40 years^33–40^. In the present study, 68.8% of patients with primary GCTs of the distal radius were diagnosed between 20 and 40 years of age (68.0% in men and 69.6% in women). Furthermore, a slightly younger age at diagnosis for GCTs of the distal radius was observed in men than in women, with a mean age at diagnosis of 28.7 years for men and of 32.6 years for women. Thus, there was a younger age at diagnosis for men among all patients with GCTs of the distal radius, but a younger age at diagnosis was previously observed in women among patients with GCT around the knee^41^. Thus, large, multicenter studies are necessary in order to determine the predominant age at GCT diagnosis in different locations in the future.

Previous studies indicated that there was a high frequency of Campanacci grade III among cases of GCT of the distal radius^37, 40^. In line with these studies, the frequency of Campanacci grade III accounted for 79.2% of cases of GCT of the distal radius in the present study, greater than that previously reported for GCT around the knee^41^. The delay in diagnosis due to the non-weight bearing location in the distal radius may contribute to the high frequency of Campanacci grade III occurring in GCTs of the distal radius.

Previous studies have indicated a great range for rates of local recurrence GCTs of the distal radius, with a rate of local recurrence between 25% and 89%^16, 39, 42–45^. In this study, we found that the prevalence of local recurrence was 30.0% overall, mildly greater in men (38.1%) than in women (21.1%).

The reported rates of pulmonary metastasis for GCTs of the distal radius have been controversial. Several studies reported that there was no pulmonary metastasis related to GCTs of the distal radius^33, 46, 47^, but a rate of 3% was reported in a different study^37^. Similar to that study, we found that the rate of pulmonary metastasis was 5.0% overall (4.8% for men and 5.3% for women). Moreover, the prevalence of pathological fractures was 12.5% in this study, and a slightly higher prevalence of fractures was observed in men (20.0%) than in women (4.3%). Compared to the reported rates for GCTs around the knee, the prevalence of fractures is lower for GCTs of the distal radius^41^.

There are limitations to this study. First, there may have been differences between the identification standards in radiological data and clinical staging at different institutions due to the multicenter retrospective study design. However, this limitation could be overcome by training the investigators from the seven centers. Moreover, all patients were recruited from 2005 to 2015; new techniques were not developed during this 15-year period, and the standard of diagnosis did not change. Second, the limited sample size may have been associated with error, but this could be improved by increasing the number of institutes involved in the GTOC.

## Conclusions

To our knowledge, this is the first report of the epidemiological and clinical characteristics of primary GCTs of the distal radius from a multicenter study in China. Consistent with the previous studies, we found a mild male predominance, greater frequency of patients aged 20–40 years and with a Campanacci grade of III, and high rates of local recurrence and pulmonary metastasis. However, we found that there was a younger age at diagnosis for men and a lower prevalence of fracture in this study compared with similar data for GCTs around the knee reported in our previous study. These findings suggest that it is crucial to evaluate and compare the demographic and clinical features of GCT in all locations and design an individualized, optimal surgical approach that balances local recurrence and functional outcome and reduces the overall disease burden of GCTs.

## Material and Methods

### Patient selection

Patient selection and the study design have been described in previous studies^20, 41^. The GTOC team is an association of physicians from seven orthopedic oncology centers located in different regions of China. Seven centers established the GTOC in 2005. The aim of the GTOC is to standardize the diagnosis and treatment of GTCs in China. The current standardized procedure was devised by GTOC experts who considered GCTs’ pathological fractures, shifting of the articular surface, Campanacci grade, the growth level of the tumor (i.e., the tumor has grown with or without breaking through the articular surface), and tumor volume.

We retrospectively reviewed all qualified patients’ medical records. All patients had received their first histologically confirmed diagnoses of benign GCT and undergone surgical treatment. We excluded all patients with a preoperative diagnosis of GCT that was postoperatively determined to have been incorrect (i.e., patients with a non-GCT postoperative diagnosis). Patients who were not treated surgically did not have their diagnosis of GCT confirmed by a pathological method. Moreover, patients with recurrent GCT received treatment more than once, and the natures of the GCTs changed in these patients due to recurrence. Thus, we further excluded all cases of GCT recurrence and all patients who were not treated surgically. Clinical and imaging data of the primary GCTs in the distal radius were also reviewed retrospectively.

In total, we recruited 51 patients with primary GCTs in the distal radius and analyzed the clinical and epidemiological features of 48 cases after excluding three patients who were <13 years old. We also assessed the rate of local recurrence for 40 cases, with a response rate of 83.3%.

### Campanacci grade

All patients with a GCT of the distal radius were stratified according to the Campanacci system, which is based on plain radiography^48^. Under the Campanacci system, grade I tumors are the least common and show features of latent or slow-growing tumors. The lesion is small, with a mild amount of sclerosis delineating the tumor. The bone contour is not affected, although the cortex can be thinned. The tumor does not extend to the articular cartilage. Symptoms are absent or minimal and of long duration. Grade II tumors show features of an active lesion with ill-defined borders and without sclerosis. The cortex is thinned, if not breached and deformed with expansion, and the periosteum is elevated. The tumor often extends to the articular cartilage from within the marrow. Grade III tumors show features of extreme aggressiveness, with a tumor that has a large volume, destroys bone, and invades the surrounding soft tissues.

### Evaluation of clinical features

All patients were routinely evaluated preoperatively with plain radiography, computed tomography (CT), and magnetic resonance imaging (MRI) scans of the involved forearm and CT of the chest, after which they were all classified according to the Campanacci staging system for GCT of bone. Additionally, serum calcium, phosphorus, and alkaline phosphatase concentrations were routinely measured to rule out hyperparathyroidism. Core needle biopsies were then obtained if the lesion was atypical of GCT clinically or radiologically.

### Surgical treatment

The surgical approach was chosen on the basis of the location of the tumor and the preference of the treating surgeon according to the GTOC’s predefined plan. The biopsy tract, if present, was removed in the initial incision. En bloc resection of the distal radius was then performed with a safe margin, after which the proximal fibula was excised as previously reported^38, 49^. The fibular graft was then placed into the forearm and fixed to the proximal radius with a compression plate or one-third tubular plate. K-wires were utilized to stabilize the newly reconstructed joints, if necessary. Next, the remnant fibular collateral ligament was sutured to the radial collateral ligaments to increase the stability of the joints.

All radiographic images of the forearm and chest were reviewed by experienced surgeons and imaging physicians to determine whether bony union, recurrence, tumor metastasis, or complications had occurred.

Denosumab was not used to treat patients postoperatively, as it was not available during the study period (2000–2014). Moreover, it has not been approved in China by the FDA. Thus, there were no additional treatments administered postoperatively.

### Follow-up and outcomes

The patients were followed every 3 months for the first 2 years post-operatively, every 6 months until 5 years post-operatively, and every 12 months until 10 years post-operatively. Telephone interviews were allowed only after 5 years of follow-up. Information related to local recurrence and metastases was obtained according to the present mass by face-to-face evaluation. Patients who experienced local recurrence or metastases for which they were treated in another hospital were interviewed by telephone.

### Statistical methods

All patient data were analyzed according to sex and age group. Because cases of GCT most commonly occur among patients 20–40 years old, age was categorized into two groups: ≤40 years and >40 years. Continuous variables were summarized in terms of means (standard deviations), and differences in age were compared using Student’s t-test after confirmation of normality. Categorical variables were summarized in terms of number of cases (percentages). The chi-squared test was used to compare differences in the clinical characteristics between sex and age groups. Categorical variables with <5 expected frequencies were compared using Fisher’s exact test. Statistical significance was defined as P < 0.05.

### Availability of data and material

The datasets used and/or analyzed during the current study are available from the corresponding author on reasonable request.

## Declarations

### Ethics approval and consent to participate

All investigative protocols were approved by the ethics committee of Tianjin Hospital. The procedures were performed according to approved guidelines, and written informed consent was obtained from each patient.
